# Clinical characteristics and molecular evolution of ST11-KL64 carbapenem-resistant hypervirulent *Klebsiella pneumoniae* co-producing KPC-2 and NDM-1 from China

**DOI:** 10.1128/spectrum.02911-25

**Published:** 2026-02-09

**Authors:** Letian Xia, Mengjiao Shi, Chenfei Li, Yaqi Zhu, Fei Jiang, Shulong Zhao, Shuang Song, Youzhen Ma, Lei Cheng, Haiquan Kang

**Affiliations:** 1 Department of Clinical Laboratory, The Second Affiliated Hospital of Xuzhou Medical University 608493 , Xuzhou, Jiangsu, People's Republic of China; 2 School of Medical Technology, Xuzhou Medical University 38044 , Xuzhou, Jiangsu, People's Republic of China; 3 Department of Medical Laboratory Technology, Feng Xian People's Hospital, Xuzhou, Jiangsu, People's Republic of China; 4 Department of Clinical Laboratory, Affiliated Hospital of Xuzhou Medical University 117910, Xuzhou, Jiangsu, People's Republic of China; 5 The Center for Clinical Research and Transformation of Pathogen Diagnosis, Xuzhou Medical University 38044 , Xuzhou, Jiangsu, People's Republic of China; 6 School of Life Sciences, Xuzhou Medical University 38044 , Xuzhou, Jiangsu, People's Republic of China; NHLS Tygerberg/Stellenbosch University, Cape Town, Western Cape, South Africa

**Keywords:** carbapenem-resistant *Klebsiella pneumoniae*, hypervirulence, KPC-2, NDM-1, molecular epidemiology, plasmid stability

## Abstract

The emergence of carbapenem-resistant hypervirulent *Klebsiella pneumoniae* (CR-hvKP), particularly strains co-producing dual carbapenemases such as *Klebsiella pneumoniae* carbapenemase (KPC) and New Delhi metallo-β-lactamase (NDM), poses a significant challenge in clinical settings. This study aimed to elucidate the clinical features, molecular epidemiology, virulence phenotype, and plasmid stability of ST11-KL64-type *K. pneumoniae* co-producing KPC-2 and NDM-1. We retrospectively analyzed 44 non-duplicate clinical isolates of KPC-2-NDM-1-carbapenem-resistant *K. pneumoniae* (K2N1-CRKP) and their corresponding clinical data from patients in a Chinese teaching hospital in 2021. Comprehensive analyses included antimicrobial susceptibility testing, pulsed-field gel electrophoresis (PFGE), whole-genome sequencing, serum killing assays, *Galleria mellonella* infection models, and plasmid stability experiments. All isolates were resistant to carbapenems and ceftazidime/avibactam. These strains belonged to the ST11-KL64-O1/O2v1 clone and showed high genetic relatedness (≥88% similarity) via PFGE. Among them, 79.55% (35/44) were classified as CR-hvKP, carrying the virulence genes *iucABCD* and *rmpA*/*rmpA2*. Genomic analysis revealed that *bla*_KPC-2_ and *bla*_NDM-1_ were located on an IncFII/IncR plasmid and an untypable plasmid, respectively, while the virulence gene cluster was identified on an IncFIB/IncHI1B virulence plasmid. The virulence phenotypes of selected strains exhibited heterogeneity. One isolate (KP1225) demonstrated high virulence *in vitro* and *in vivo*. Notably, these strains maintained their resistance plasmids without antibiotic pressure (retention rate >85% after 10 days), with no significant fitness cost observed. This study reveals a clonal dissemination of ST11-KL64-K2N1-CR-hvKP, successfully integrating carbapenem resistance and hypervirulence with high genetic stability. The potential risk of its spread warrants urgent attention and effective infection control measures.

**IMPORTANCE:**

The emergence of bacterial pathogens that combine hypervirulence with advanced antimicrobial resistance poses a significant challenge for modern healthcare. This study comprehensively analyzes a concerning clinical strain: ST11-KL64 *K. pneumoniae* co-harboring both KPC-2 and NDM-1 carbapenemases. We demonstrate that this strain successfully integrates multiple resistance mechanisms, hypervirulence determinants, and remarkable genetic stability without observable fitness costs—a combination that significantly complicates treatment and infection control. Critically, the stable maintenance of its resistance plasmids without antibiotic selection highlights the potential for persistent colonization and transmission. These findings underscore an evolving threat in the landscape of multidrug-resistant infections and emphasize the urgent need for enhanced surveillance, robust diagnostic approaches, and novel therapeutic strategies to address such challenging pathogens.

## INTRODUCTION

Carbapenem-resistant *Klebsiella pneumoniae* (CRKP) has emerged as one of the most challenging public health threats worldwide. Its resistance is primarily mediated by carbapenemases, which hydrolyze carbapenems, third-generation cephalosporins, monobactams, and other β-lactam antibiotics, rendering conventional therapeutic options largely ineffective. CRKP infections are associated with prolonged hospital stays, increased healthcare costs, and elevated mortality rates, particularly among immunocompromised or critically ill patients with multiple comorbidities (1, 2).

Among Enterobacteriaceae, the production of carbapenemases is the core mechanism underlying carbapenem resistance. In China, CRKP strains carrying *bla*_KPC_ and *bla*_NDM_ are predominant, and the prevalence of *Klebsiella pneumoniae* carbapenemase (KPC) and New Delhi metallo-β-lactamase (NDM) co-producing CRKP (KN-CRKP) has rapidly increased (3). KPC enzymes belong to the Ambler class A serine β-lactamases and can be inhibited by the novel β-lactamase inhibitor avibactam. Hence, ceftazidime-avibactam has become a first-line agent for treating KPC-producing CRKP infections (4–6). In contrast, NDM metallo-β-lactamases (class B) are not susceptible to currently available β-lactamase inhibitors, further narrowing therapeutic options (7). The proportion of KN-CRKP isolates in China has increased from 0% in 2010 to 4.4% in 2021 (3). Notably, patients with KN-CRKP bloodstream infections exhibit a 30-day mortality rate as high as 56.0%, significantly exceeding the 32.5% rate observed in those infected with KPC-producing CRKP alone (8). The synergistic activity of these two carbapenemases not only enhances resistance to carbapenems but may also extend the spectrum of resistance to other β-lactams through complementary substrate profiles, posing serious challenges to antimicrobial stewardship.

Globally, 16 subtypes of KN-CRKP have been identified. The most prevalent are K2N1-CRKP (53.9%, carrying *bla*_KPC-2_ and *bla*_NDM-1_), K2N5-CRKP (18.8%), and K3N1-CRKP (14.9%). In China, K2N1-CRKP (62.9%) and K2N5-CRKP (35.3%) dominate, whereas K3N1-CRKP (37.1%) and K2N1-CRKP (17.9%) are more common in the United States, indicating significant geographic variation (9). Of particular concern is the recent emergence of carbapenem-resistant hypervirulent *K. pneumoniae* (CR-hvKP), which blurs the historical distinction between classical *K. pneumoniae* (cKP) and hypervirulent *K. pneumoniae* (hvKP). Moreover, the detection rate of CR-hvKP carrying the KL64 capsular locus in China has increased steadily from 28.2% in 2016 to 45.7% in 2020 (10). Studies indicate that ST11 CRKP is the most prevalent sequence type among KN-CRKP isolates (40.8%), with KL64 and O1/O2v1 lipopolysaccharide loci accounting for 47.6% and 42.4%, respectively. Notably, 95.2% of these isolates originated in China (3). The convergence of hypervirulence and dual carbapenemase resistance in KN-CRKP significantly heightens the risk of outbreaks and poor clinical outcomes, underscoring the urgent need for sustained surveillance and precise interventions (11).

Although sporadic reports have indicated the coexistence of KPC and NDM in individual CRKP strains, most studies have been limited to case reports or tiny sample sets. A systematic investigation into the clinical infection spectrum, co-evolution of resistance and virulence, and plasmid stability is lacking, hindering a comprehensive understanding of their molecular evolution and epidemiological traits. Therefore, this study established a relatively large single-center cohort of K2N1-CRKP isolates and, for the first time, integrated whole-genome sequencing (WGS), plasmid stability kinetics, and longitudinal clinical data to systematically elucidate their clonal dissemination networks, vertical/horizontal plasmid transfer efficiency, and co-carriage patterns of resistance and virulence genes. Our findings provide critical evidence for deciphering the molecular evolution of dual-carbapenemase-producing CRKP, optimizing infection control strategies, and developing targeted interventions. These insights are essential for curbing the global spread of such high-risk “hypervirulent-dual-resistant” clones.

## MATERIALS AND METHODS

### Strain collection and identification

This single-center, retrospective cohort study included 44 non-duplicate K2N1-CRKP isolates obtained from initial clinical samples collected between January and December 2021 at a teaching hospital in China. Species identification was performed using matrix-assisted laser desorption/ionization time-of-flight mass spectrometry (EXS3000, Zhongyuan Huiji, Chongqing, China). Antimicrobial susceptibility testing (AST) was carried out using the VITEK-2 Compact GN-AST card (bioMérieux, France), and results were interpreted according to the Clinical and Laboratory Standards Institute guidelines (2021). CRKP was defined as exhibiting a minimum inhibitory concentration (MIC) ≥4 mg/L for either imipenem or meropenem. *K. pneumoniae* ATCC 700603 was used as the quality control strain.

Corresponding clinical data were collected for each patient, including sex, age, ward type, comorbidities (assessed via the Charlson comorbidity index), ICU admission status, invasive procedures (mechanical ventilation, central venous catheterization, urinary catheterization, surgical history, etc.), history of antimicrobial therapy, and 30-day outcome (survival or death). Two researchers independently extracted and cross-verified all data to ensure completeness and consistency.

### WGS and bioinformatics analysis

Genomic DNA was extracted from all 44 isolates using a Bacterial Genomic DNA Rapid Extraction Kit (Sangon Biotech, Shanghai, China). Subsequent WGS was performed on the Illumina NovaSeq X Plus platform (Novogene, Beijing, China). Raw sequencing data were *de novo-*assembled using Unicycler v0.5.0 to generate draft genomes.

Assembled genomes were annotated for antimicrobial resistance genes via the CARD Resistance Gene Identifier online tool (http://proksee.ca). Further analyses were conducted using the BacWGSTdb database (http://bacdb.cn/BacWGSTdb/Tools.php), including the following: prediction of antimicrobial resistance and virulence genes; multilocus sequence typing (MLST) based on seven housekeeping genes (*gapA*, *infB*, *mdh*, *pgi*, *phoE*, *rpoB*, and *tonB*); and phylogenetic tree construction based on single-nucleotide polymorphisms (SNPs). The phylogenetic tree was visualized using iTOL (https://itol.embl.de/). Capsular serotype (K-type) and lipopolysaccharide antigen (O-type) were determined using Kleborate.

### Homology analysis

Pulsed-field gel electrophoresis (PFGE) was performed on all isolates to assess their clonal relatedness (12, 13). Fresh bacterial colonies cultured for 48 h were embedded in low-melting-point agarose plugs and lysed with proteinase K to extract intact genomic DNA. The DNA was digested with 40 U/μL of XbaI (Takara) at 37°C for 2 h in a 100 μL reaction system. The resulting fragments were separated in 0.5× TBE buffer using the CHEF Mapper XA System (Bio-Rad, USA) under the following conditions: voltage 6 V/cm, included angle 120°, pulse time 2.16–63.8 s, temperature 14°C, and run time 18 h. Salmonella Braenderup H9812 was used as the molecular weight marker (14). After electrophoresis, the gel was stained with GELRed and visualized using a Gel Doc XR+ imaging system (Bio-Rad).

PFGE profiles were normalized and analyzed with Quantity One v4.6.9. Band matching and similarity calculation were performed using the Dice coefficient, and a dendrogram was generated via the unweighted pair group method with arithmetic mean (UPGMA). A position tolerance of 1.5% and an optimization coefficient of 1.0% were applied. Isolates with ≥85% similarity were considered to belong to the same PFGE clonal type (15, 16).

### WGS, assembly, and annotation

Based on the next-generation sequencing (NGS) results, three representative isolates (KP1225, KP1303, and KP1441) were selected for full-length WGS. These three isolates were distributed across different branches of the phylogenetic tree construction based on SNPs, representing minor but discernible genetic variations within the clone, and they were isolated from different wards and time points. Bacterial genomic DNA extraction and assembly methods followed the procedures described in the NGS section. Sequencing was performed using the PacBio Sequel II platform (Novogene, Beijing, China). Genome annotation was conducted using the Bakta tool available at http://proksee.ca, and plasmid sequences were aligned using BLAST. Relevant reference plasmid sequences were retrieved from the NCBI database (https://www.ncbi.nlm.nih.gov/) and compared with other known plasmid sequences.

Comparative genomic and collinearity analyses of plasmids were performed using Easyfig v2.2.5. Sequence alignment was carried out with BLAST v2.9.0, applying an identity threshold of ≥80% and filtering out minor matched regions and matches shorter than 1 kbp. Final figures were edited and integrated using Adobe Illustrator 2025. The complete genome sequences of KP1225, KP1303, and KP1441 have been deposited in the NCBI GenBank database under the BioProject accessions PRJNA1292292, PRJNA1292749, and PRJNA1292752, respectively.

### Serum killing assay

The three selected isolates (KP1225, KP1303, and KP1441) were subjected to a serum killing assay, as previously described (17, 18). Serum was collected from 10 healthy volunteers, pooled, aliquoted, and stored at –80°C until use. Bacterial suspensions in the mid-log phase were adjusted to 1 × 10⁶ CFU/mL in phosphate-buffered saline (PBS) and mixed with the pooled human serum at a 1:3 volume ratio. Samples were taken at 0, 1, 2, and 3 h, plated on Mueller-Hinton agar, and colony counts were taken. Serum sensitivity was classified into levels 1–2: serum sensitivity, 3–4 intermediate sensitivity, and 5–6 resistance. All experiments were independently repeated three times. *Klebsiella pneumoniae* ATCC 43816 and ATCC 700603 were used as high-virulence and low-virulence control strains, respectively (16, 19, 20).

### Pathogenicity assessment using a *Galleria mellonella* infection model

To further evaluate the *in vivo* pathogenicity of the isolates, a *G. mellonella* infection model was employed as described previously (21–23). The same test and control strains used in the serum killing assay were evaluated. Healthy *G. mellonella* larvae weighing 250–350 mg were selected for the experiments.

Bacterial suspensions from the logarithmic growth phase were adjusted to concentrations ranging from 1 × 10⁵ to 1 × 10⁸ CFU/mL using PBS. For each group, 10 larvae were injected with 10 µL of the bacterial suspension into the last proleg. After injection, each larva was placed individually in a sterile petri dish and incubated at 37°C in the dark. Survival was monitored over 72 h. All infection experiments were performed in three independent replicates.

### Fitness cost and plasmid stability assays

A total of eight *K. pneumoniae* strains were used, including six clinical isolates co-harboring *bla*_KPC-2_ and *bla*_NDM-1_ (KP1137, KP1225, KP1296, KP1316, KP1434, and KP1436), along with control strains ATCC BAA-1705 (producing only KPC-2) and ATCC BAA-2146 (producing only NDM-1). All strains were inoculated onto LB agar and cultured at 37°C for 18 h. Single colonies were then transferred into 5 mL of LB broth and incubated at 37°C with shaking at 200 rpm for 18 h. The bacterial suspensions were adjusted to an OD600 of 0.01, and 500 µL was inoculated into 50 mL of fresh LB broth; blank LB medium served as a negative control. Growth was monitored by measuring the OD600 hourly to generate growth curves.

Plasmid stability was evaluated as described previously (24) with minor modifications. The 6 test isolates were inoculated into 5 mL of LB broth containing imipenem (3 mg/L) and cultured at 37°C with shaking at 200 rpm for 18 h. Then, 1 mL of culture was adjusted to an OD600 of 1 and subcultured at a 1:1,000 ratio into 5 mL of fresh LB broth with imipenem (3 mg/L) for another 18 h. Subsequently, 1 mL of this culture was adjusted to an OD600 of 0.1 and subcultured again at 1:1,000 into 5 mL of antibiotic-free LB broth. This passage was repeated daily for 10 days under the same conditions.

After 10 days, the final cultures were serially diluted and plated onto antibiotic-free LB agar and LB agar containing imipenem (3 mg/L). The plasmid retention rate was calculated as the ratio of colony counts on imipenem-containing plates to those on antibiotic-free plates. On day 10, 20 randomly selected colonies were subjected to PCR to confirm the presence of *bla*_KPC-2_ and *bla*_NDM-1_, and their susceptibility to meropenem and ceftazidime/avibactam was assessed using the disk diffusion method.

## RESULTS

### Clinical characteristics of patients

Clinical data were collected from 44 patients infected with K2N1-CRKP (Table 1). The cohort included 32 males and 12 females, all adults aged 27 to 84 years. The majority of isolates were obtained from respiratory sources, with sputum specimens accounting for 40 cases (90.91%), followed by urine (3 cases, 6.82%) and blood (1 case, 2.27%). Underlying comorbidities were present in 37 patients (84.09%), including neurological, cardiovascular, and urinary system diseases, as well as diabetes and hypertension. A history of traumatic injury was reported in 12 patients (27.27%). Most patients (97.73%, 43/44) had undergone invasive procedures such as surgery, central venous catheterization, urinary catheterization, or endotracheal intubation.

**TABLE 1:** Patients’ clinical features^*a*^

Isolates	Sex	Age	Ward	Underlying diseases	Antibiotic history	Invasive procedures	Treatment	Sample type	Isolate time	Outcome
KP1035	M	50	URS	EP, UTI, and urolithiasis	CC	Surgery and UC	TCF	UR	2021/1/5	Survived
KP1037	M	77	NES	CH and HTN	AK and BIA	Surgery and UC	AK and TGC	SP	2021/1/8	Survived
KP1100	F	47	NES	CH	MEM and CTS	Surgery, TC, and UC	TGC and BIA	SP	2021/1/28	Died
KP1111	M	69	NES	CH, CI, and lung	CTS and MEM	Surgery, TC, and UC	TCF and BIA	SP	2021/1/30	Survived
KP1128	M	78	ICU	Esophageal cancer and HTN	IPM and TGC	Surgery, TC, and UC	IPM and BIA	UR	2021/2/18	Survived
KP1137	M	43	EICU	Liver rupture, fracture, SEP, and ARDS	TGC and AK	Surgery, TC, UC, and CVC	CID and MXF	SP	2021/2/22	Survived
KP1160	M	63	ICU	Uremia, HTN, and DM	TCF	TC and UC	CTS and TCF	SP	2021/3/8	Died
KP1161	M	44	ICU	CH and HTN	MEM	Surgery, TC, UC, and CVC	LEV, IPM, SCF, and TGC	SP	2021/3/9	Died
KP1189	F	31	ICU	CH, HTN, diabetes insipidus, and SEP	CDZ	Surgery, TC, UC, and CVC	TCF	SP	2021/3/21	Died
KP1190	M	62	NES	CI, heart disease, hyperthyroidism, and SEP	TCF and MXF	Surgery, TC, UC, and CVC	MXF and SCF	SP	2021/3/22	Survived
KP1196	F	76	ICU	CH and heart disease	MEM, TCF, and ATM	Surgery, TC, UC, and CVC	VA, MEM, TCF, and CDZ	SP	2021/3/25	Died
KP1197	M	65	NSICU	CH, SEP, CI, and heart disease	VA and ATM	Surgery, TC, UC, and CVC	TGC and BIA	SP	2021/3/26	Died
KP1209	M	69	NES	CH and fracture	LEV and SCF	Surgery	LEV	SP	2021/3/23	Survived
KP1216	M	52	NES	CH and fracture	TGC and FEP	Surgery, TC, and UC	MSU	SP	2021/3/30	Survived
KP1222	M	47	EICU	CH, HTN, SEP, and ARDS	CTS	TC, UC, and CVC	MSU	SP	2021/4/1	Died
KP1223	M	64	NES	CH, fracture, SEP, and GI-Bleed	CSL and BIA	Surgery, TC, UC, CVC	BIA, AK, PB, CAZ, and MXF	SP	2021/4/1	Survived
KP1225	M	47	NSICU	CI, HTN, SEP, and DVT	BIA and FEP	Surgery, TC, UC, and CVC	BIA and CFP	SP	2021/4/1	Died
KP1239	M	77	NES	CH, SEP, and liver dysfunction	CRO and BIA	Surgery, TC, UC, and CVC	BIA	SP	2021/4/7	Died
KP1240	M	80	NES	CH, SEP, HTN, and DM	TCF and CID	Surgery and UC	CFP, MXF, and BIA	SP	2021/4/7	Died
KP1272	M	76	EICU	CH, HTN, SEP, and DM	AK and TZP	TC and UC	AK and TGC	SP	2021/5/3	Died
KP1289	M	77	GES	HTN, colon cancer, CI, and heart disease	IPM and SCF	Surgery, UC, and CVC	IPM	SP	2021/5/10	Died
KP1296	M	81	HEM	MDS, SEP, liver dysfunction, and ARDS	MXF and SCF	/^*b*^	MXF and SCF	SP	2021/5/12	Died
KP1303	F	73	EICU	CH, SEP, DVT, and PE	CTS	Surgery and UC	CTS, MSU, AK, and TGC	SP	2021/5/18	Died
KP1306	F	71	EICU	CH, HTN, and SEP	SCF and LEV	Surgery, TC, and UC	SCF, LEV, and IPM	SP	2021/5/19	Survived
KP1316	F	70	EICU	CH, HTN, and DM	CID	Surgery, TC, UC, and CVC	TGC, MEM, VA, and SCF	SP	2021/5/26	Died
KP1336	M	77	NICU	CH, fracture, HTN, and ELD	CTS	Surgery, TC, and UC	CTS and LEV	SP	2021/6/5	Died
KP1349	M	66	NICU	Fracture and multiple organ dysfunction	CSL and LNZ	TC, UC, and CVC	CSL, ET, and AK	SP	2021/6/15	Survived
KP1353	M	71	NICU	CH, fracture, HTN, CI, and ELD	CRO	UC	CRO and TGC	SP	2021/6/21	Died
KP1359	M	64	NICU	CI, EP, ELD, and HTN	CSL	TC and UC	CSL, TGC, and CTS	SP	2021/6/28	Died
KP1363	M	63	NICU	CH and fracture	CID and CSL	Surgery and UC	MXF and CTX	SP	2021/6/29	Survived
KP1366	M	70	NICU	CH, ICI, HBV, HTN, and DVT	CRO and ET	TC and UC	ET, BIA, TGC, and CTS	SP	2021/7/2	Survived
KP1377	M	56	NICU	CI, HTN, and DM	SCF and LNZ	UC and CVC	CTS	SP	2021/7/2	Survived
KP1389	F	64	NICU	CH, fracture, SEP, and HTN	CTS and CAZ	TC, UC, and CVC	CAZ, TGC, and AK	BL	2021/7/15	Died
KP1398	M	84	NICU	CI, HTN, and SEP	CSL	TC, UC, and CVC	MEM and TGC	SP	2021/7/15	Died
KP1403	M	27	EICU	ICI and SEP	AK and TGC	UC	AK and TGC	UR	2021/7/19	Died
KP1409	M	66	NICU	CI, SEP, HTN, and PE	MEM, TGC, and CSL	TC, UC, and CVC	MEM, TGC, and AK	SP	2021/7/24	Survived
KP1425	M	80	EICU	ACS, HTN, CI, and renal pelvic cancer	TCF	TC, UC, and CVC	TGC and CAZ	SP	2021/8/4	Died
KP1426	F	71	EICU	UTI, CI, DM, HTN, and heart disease	IPM and LEV	TC, UC, and CVC	IPM, MXF, and TGC	SP	2021/8/4	Died
KP1430	F	60	NICU	Fracture	CID	Surgery, TC, UC, and CVC	IPM, LNZ, and TGC	SP	2021/8/9	Died
KP1434	F	71	EICU	CI, PE, ARDS, DVT, DM, HTN, and heart disease	TCF	TC, UC, and CVC	TCF and MEM	SP	2021/8/13	Died
KP1436	F	71	NEU	Tetanus, CI, SEP, HTN, and heart disease	SCF	TC, UC, and CVC	SCF and IPM	SP	2021/8/14	Survived
KP1441	M	75	NEU	CH, SEP, CI, and DVT	BIA and LNZ	UC	BIA, LNZ, TCF, and MXF	SP	2021/8/18	Died
KP1443	M	73	NES	CH, HTN, DM, and SEP	SCF and TGC	Surgery, TC, UC, and CVC	SCF, LEV, TGC, AK, and CAZ	SP	2021/8/21	Survived
KP1540	F	63	NICU	CH and SEP	TCF	UC	CAZ, ET, and PB	SP	2021/12/28	Survived

^*a*^ M, male; F, female; URS, urological surgical department; NES, neurosurgery; ICU, intensive care unit; EICU, emergency intensive care unit; NSICU, neurosurgical intensive care unit; GES, general surgical department; HEM, hematology department; NICU, neurology intensive care unit; NEU, neurology; EP, epilepsy; UTI, urinary tract infection; CH, cerebral hemorrhage; HTN, hypertension; CI, cerebral infarction; SEP, severe pneumonia; ARDS, acute respiratory distress syndrome; DM, diabetes; GI-Bleed, gastrointestinal bleeding; DVT, deep vein thrombosis; MDS, myelodysplastic syndrome; PE, pulmonary embolism; ELD, electrolyte disturbance; ICI, intracranial infection; HBV, hepatitis B; ACS, acute coronary syndrome; UC, urinary cannula; TC, trachea cannula; CVC, central venous cannula; ATM, aztreonam; AK, amikacin; BIA, biapenem; CAZ, ceftazidime; CDZ, cefodizime; CFP, cefepime; CID, cefonicid; CRO, ceftriaxone; CSL, cefoselis; CTS, cefotaxime/sulbactam; CTX, cefotaxime; ET, etimicin; IPM, imipenem; LEV, levofloxacin; LNZ, linezolid; MEM, meropenem; MSU, melocillin/sulbactam; MXF, moxifloxacin; PB, polymyxin B; SCF, cefoperazone/sulbactam; TCF, cefoperazone/tazobactam; TGC, tigecycline; TZP, piperacillin/tazobactam; VA, vancomycin; UR, urine; SP, sputum; BL, blood.

^*b*^ "/" indicates that the patient did not undergo any invasive procedures.

In terms of department distribution, 12 cases (27.27%) were from the NICU, with 11 of these isolates obtained between 5 June and 9 August 2021; 10 cases (22.73%) were from the NEU department, including six isolates collected within 16 days (22 March to 7 April 2021); 10 cases (22.73%) were from the EICU, with four isolates detected in May 2021 and another four concentrated between 19 July and 13 August 2021; and 5 cases (11.36%) were from the general ICU, with isolates obtained between 18 February and 25 March 2021. The Gantt chart clearly demonstrates the temporal clustering of cases within specific wards (NICU/EICU), providing strong circumstantial evidence for independent clonal transmission events prior to the implementation of enhanced infection control measures (Fig. S1).

### Strain homogeneity analysis

**Fig 1:**
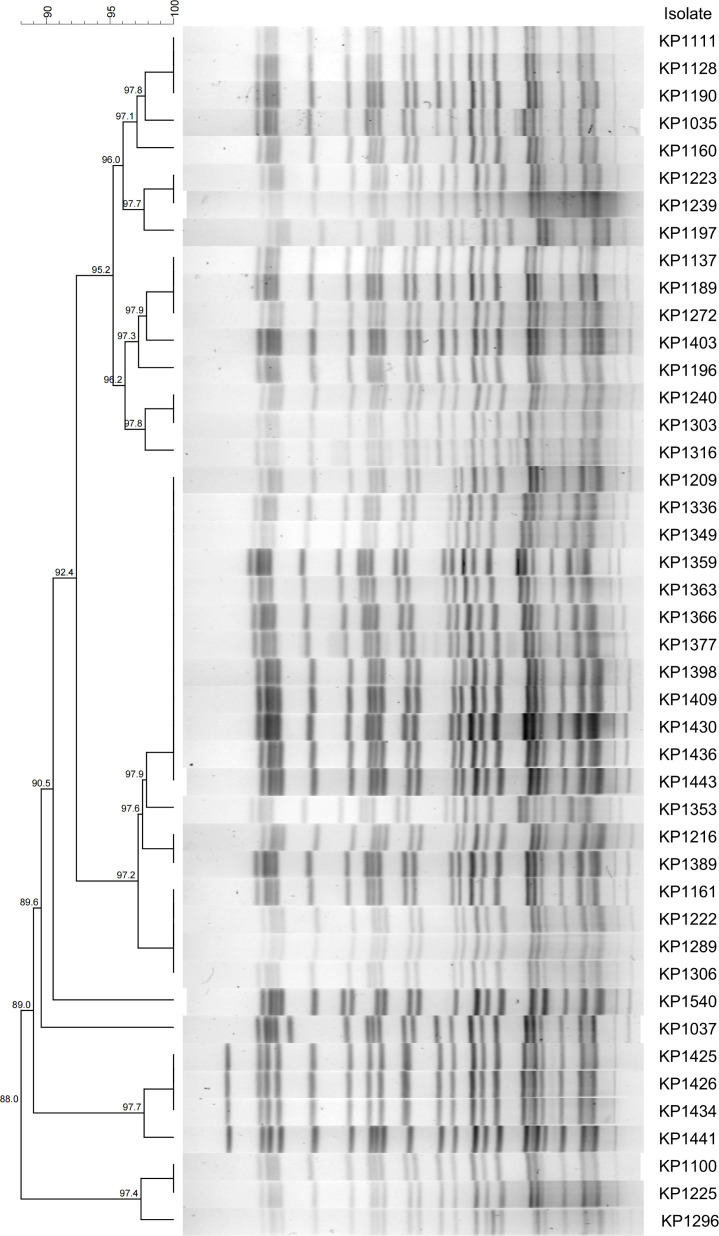
PFGE profile analysis of 44 strains of KPC-2-NDM-1-CRKP. A dendrogram was constructed using the UPGMA algorithm based on Dice similarity coefficients. Isolates were considered to belong to the same clone cluster when their Dice similarity index exceeded 85%.

MLST based on seven housekeeping genes revealed that all isolates shared an identical allelic profile: *gapA 3*, *infB 3*, *mdH 1*, *pgI 1*, *phoE 1*, *rpoB 1*, and *tonB 4*, corresponding to sequence type ST11. This sequence type is the most prevalent among clinical *K. pneumoniae* isolates in China. PFGE demonstrated no less than 88% genetic homology among all strains (Fig. 1). Combined with phylogenetic analysis, these 44 isolates were closely related and could be classified into the same clonal cluster, indicating that they likely originated from a single clonal transmission event.

### Antimicrobial susceptibility and resistance gene profiles

**Fig 2:**
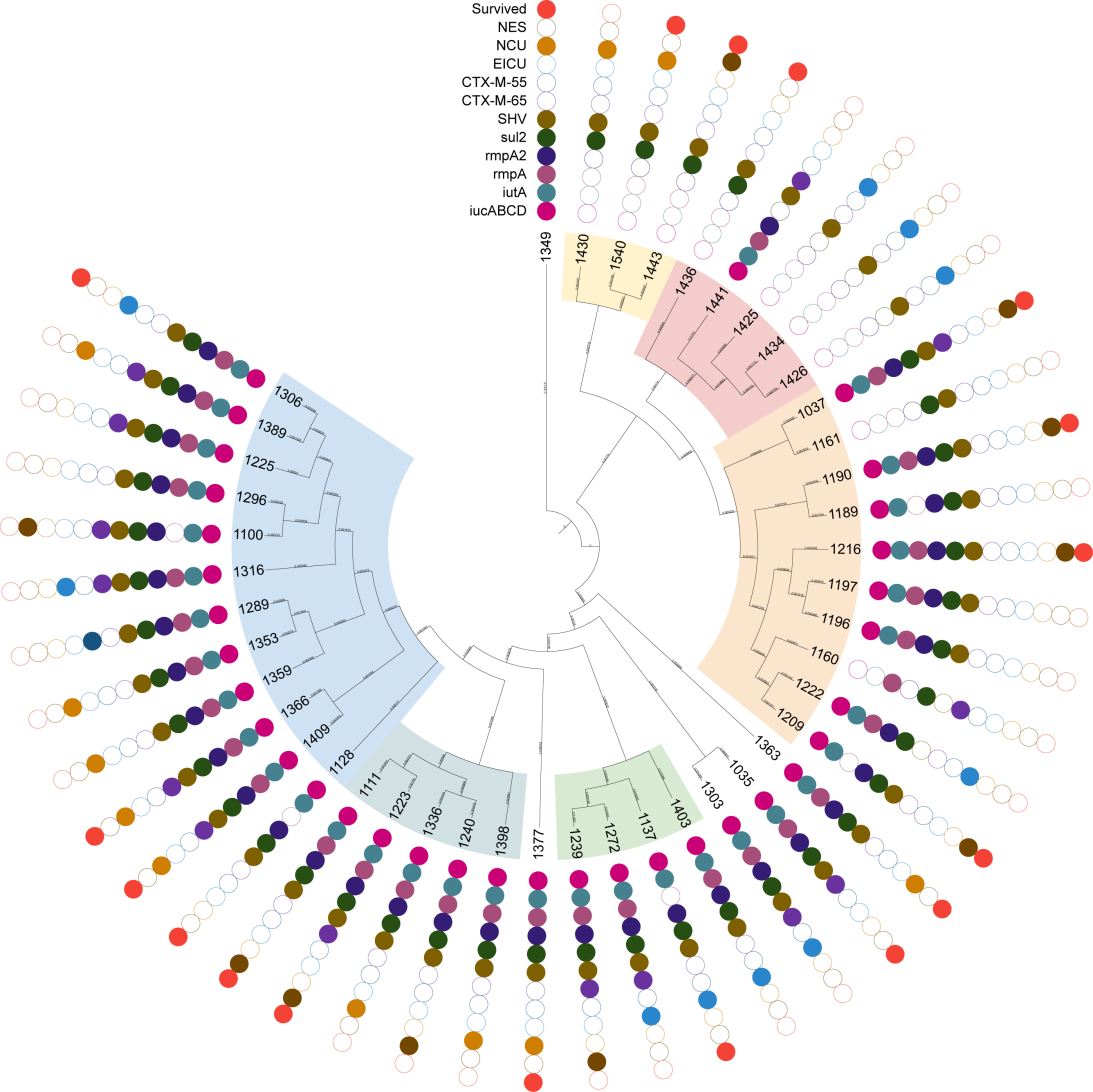
Phylogenetic analyses of 44 *K*. *pneumoniae* strains co-carrying *bla*_KPC_ and *bla*_NDM_. The outer circle indicates whether the patient survived. Cells of various colors in the inner 11 circles indicate wards with various antimicrobial resistance genes or virulence genes. In contrast, blank cells indicate death or the absence of the gene.

AST revealed that all isolates exhibited high-level resistance to penicillins, cephalosporins, and carbapenems, with varying degrees of resistance to ceftazidime/avibactam (Table 2). Genetic analysis confirmed the presence of *bla*_NDM-1_, *bla*_KPC-2_, *dfrA*1*4*, *fosA*, *LAP-2*, *QnrS1*, *rmtB*, and *TEM-1* in all isolates. The SHV gene was detected in 42 strains, while 15 isolates carried either *bla*_CTX-M-65_ or *bla*_CTX-M-55_ (Fig. 2). Notably, isolate KP1239 exhibited high-level resistance to colistin, and KP1425 was resistant to TGC.

**TABLE 2:** Antibiotic resistance characteristics^*a*^

Characteristics		*n* (%)	Isolates^*b*^
Drug	MIC or diam		
IPM	≥16	44 (100.00)	
MEM	≥16	44 (100.00)	
TZP	≥128	44 (100.00)	
AK	≥64	44 (100.00)	
ATM	≥64	44 (100.00)	
CFP	≥32	44 (100.00)	
CTX	≥64	44 (100.00)	
CAZ	≥64	44 (100.00)	
CIP	≥4	44 (100.00)	
SXT	≥320	40 (90.91)	
	40	2 (4.55)	KP1425/KP1441
	160	1 (2.27)	KP1426
	80	1 (2.27)	KP1434
POL	≤0.5	43 (97.73)	
	≥16	1 (2.27)	KP1239
TGC	2	43 (97.73)	
	≥8	1 (2.27)	KP1441
CZA	10	3 (6.82)	
	11	2 (4.55)	
	12	7 (15.91)	
	13	2 (4.55)	
	14	10 (22.73)	
	15	11 (25.00)	
	16	6 (13.64)	
	18	1 (2.27)	
	19	2 (4.55)	

^*a*^ CIP, ciprofloxacin; SXT, cotrimoxazole; POL, polymyxin; CZA, ceftazidime/avibactam. VITEK-2 Compact automatic microbial identification drug sensitivity analyzer (Merieux, France) was used for drug sensitivity detection; the unit of minimum inhibitory concentration is mg/L. CZA was detected by the KB method, and the diameter unit of the antibacterial zone was mm.

^*b*^ Empty cells denote the absence of a designated strain.

Isolates KP1425 and KP1441 were susceptible to trimethoprim-sulfamethoxazole (SXT), consistent with the absence of the sul2 gene. Interestingly, KP1426 and KP1434, which also lacked sul2, were resistant to SXT, suggesting alternative resistance mechanisms. PFGE analysis showed 100% homology among KP1425, KP1426, and KP1434 and 97.7% homology with KP1441. Clinically, the patients infected with KP1425, KP1426, and KP1434 had all been hospitalized in the EICU, while the patient infected with KP1441 was transferred to the neurology ward after a 4-day stay in the EICU. The high genetic relatedness among these isolates and their epidemiological links strongly suggest clonal transmission within the EICU.

### Capsular serotyping and virulence gene profiles

All isolates belonged to capsular serotype KL64 and lipopolysaccharide antigen type O1/O2v1. Based on WGS assembly and annotation, the following virulence-associated genes were universally present: *entB*, *fimH*, *iroE*, *mrkD*, and *ybtS*. Additional virulence genes were detected at the following frequencies: *rmpA2*, *iucABCD*, and *iutA* were present in 79.55% (35/44) of isolates, and *rmpA* was found in 70.45% (31/44). Per established criteria (25, 26), strains co-harboring *iucABCD* and *rmpA*/*rmpA2* were classified as CR-hvKP. Thus, 35 isolates in this study were categorized as CR-hvKP.

Furthermore, the type VI secretion system (T6SS), a multi-subunit protein complex prevalent in gram-negative bacteria, is typically encoded by 13 core genes (tssA–tssM) and plays a key role in bacterial interactions and virulence, often associated with hypervirulent phenotypes in CRKP (27). All 44 isolates in this study encoded 11 T6SS core components: *tssA*, *tssC*, *tssD*, *tssF*, *tssG*, *tssH*, *tssI*, *tssJ*, *tssK*, *tssL*, and *tssM*.

### *In vitro* and *in vivo* pathogenicity assessment

**Fig 3:**
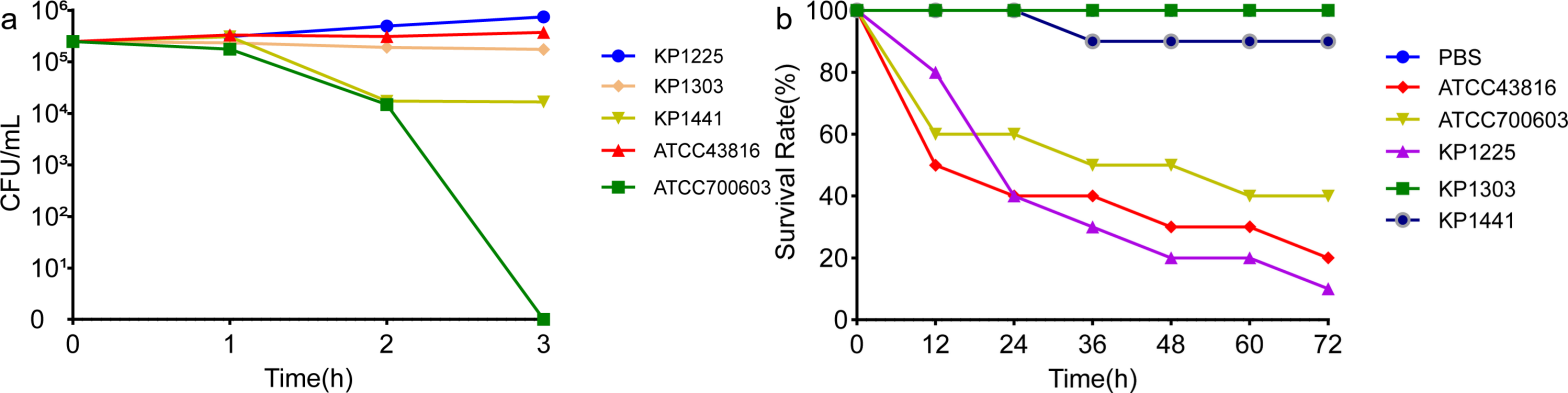
The results of *in vivo* and *in vitro* pathogenicity assessment. (**a**) Serum killing assay of three selected isolates. ATCC43816 and ATCC700603 were hypervirulent and low virulence controls, respectively. (**b**) Survival rate of *G. mellonella* larvae infected with 1 × 10^6^ CFU/mL of each selected isolate. ATCC43816 and ATCC700603 were hypervirulence and low virulence controls, respectively. PBS was the experimental control.

In the serum killing assay (Fig. 3a), KP1225 and the hypervirulent reference strain ATCC 43816 were resistant to killing by healthy human serum (Grade 6). KP1303 showed intermediate susceptibility (Grade 3), while KP1441 and the classical *K. pneumoniae* reference strain ATCC 700603 were susceptible to serum killing (Grade 1/2).

In the *G. mellonella* larval infection model (Fig. 3b), survival rates were evaluated at 72 h post-infection. At an inoculum concentration of 1 × 10⁶ CFU/mL, the survival rates were 40% for the cKP strain ATCC 700603 and 20% for the hypervirulent control ATCC 43816. In contrast, the survival rates for KP1225, KP1303, and KP1441 were 10%, 100%, and 90%, respectively, at 72 h.

### WGS analysis results

WGS of the three strains (KP1225, KP1303, and KP1441) revealed that virulence genes (including *iucABCD*, *rmpA*, and *rmpA2*) and carbapenemase genes (*bla*_KPC-2_ and *bla*_NDM-1_) were located on distinct plasmids.

**Fig 4:**
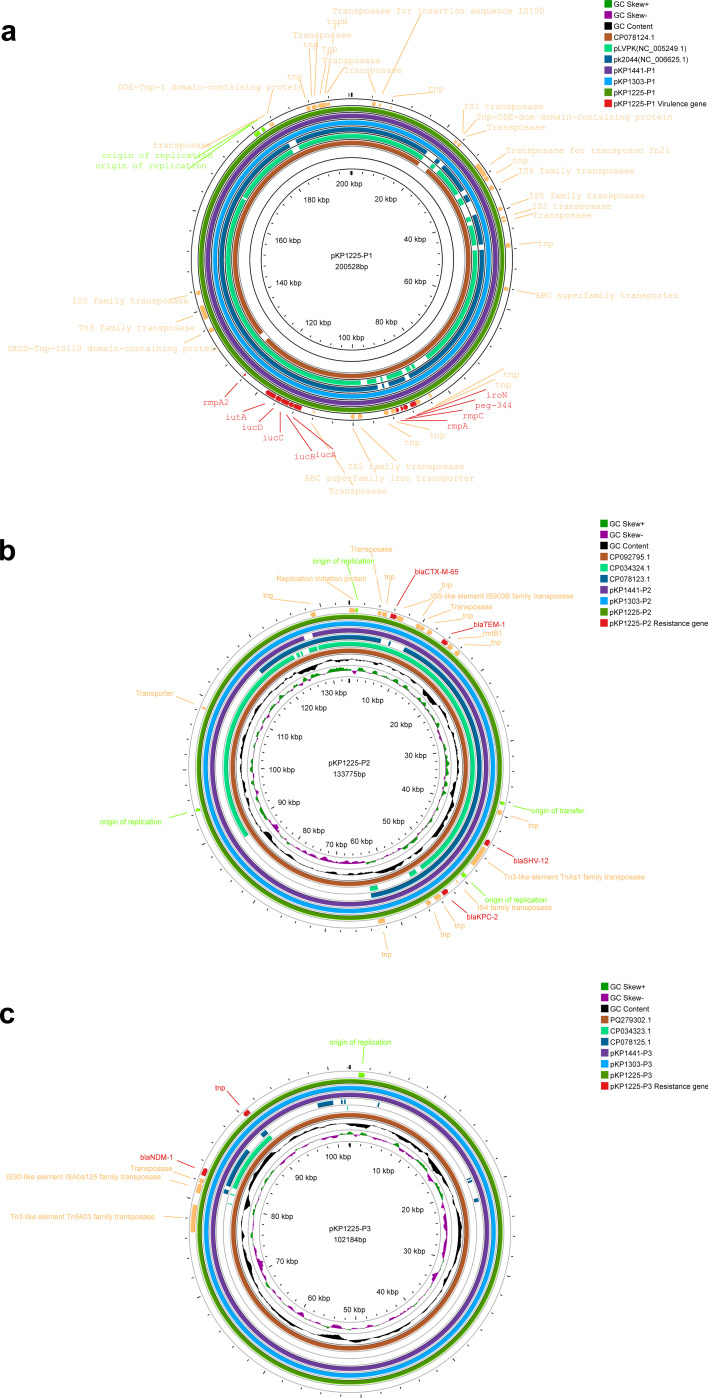
Three plasmid circle maps for genetic comparison. (**a**) Comparison of virulence gene plasmids. (**b**) Comparison of *bla*_KPC-2_ plasmids. (**c**) Comparison of *bla*_NDM-1_ plasmids. KP1225 was used as the reference plasmid.

Taking strain KP1225 as an example, it contains one circular chromosome of 5,522,891 bp and three plasmids (200,528 bp, 133,775 bp, and 102,184 bp, respectively) (Table 3). Among these, plasmid pKP1225-P1 belongs to the IncFIB/IncHI1B type and carries the virulence gene cluster *iucABCD*, *iutA*, *rmpA*, *rmpA2*, *rmpC*, *iroN*, and *peg-344*, but no antimicrobial resistance genes were detected. This plasmid exhibited 88% coverage and 99.42% sequence identity with the known virulence plasmid pLVPK (NC_005249.1) and 94% coverage with 100% identity to pK2044 (NC_006625.1) (Fig. 4a). pKP1225-P1 showed high similarity to pKP1303-P1 and pKP1441-P1, with coverage exceeding 100% and identity above 99.96%.

**TABLE 3:** Plasmid information of the strains

Isolates	Chromosome	Plasmid size			Plasmid type		
		Virulence gene	*bla*_KPC-2_	*bla*_NDM-1_	Virulence gene	*bla*_KPC-2_	*bla*_NDM-1_
KP1225	5,522,891 bp	200,528 bp	133,775 bp	102,184 bp	IncFIB/IncHI1B	IncFII(pHN7A8)/IncR	/^*a*^
KP1303	5,522,001 bp	200,503 bp	134,507 bp	102,169 bp	IncFIB/IncHI1B	IncFII(pHN7A8)/IncR	/
KP1441	5,444,881 bp	201,247 bp	133,081 bp	105,292 bp	IncFIB/IncHI1B	IncFII(pHN7A8)/IncR	/

^*a*^ “/” indicates that the strains could not be assigned to any known plasmid replicon type using PlasmidFinder.

In contrast, *bla*_KPC-2_ and *bla*_NDM-1_ were located on two different plasmids: pKP1225-P2 and pKP1225-P3, respectively. pKP1225-P2 carries multiple β-lactamase genes, including *bla*_KPC-2_, *bla*_TEM-1_, *bla*_CTX-M-65_, and *bla*_SHV-12_ (Fig. 4b). This plasmid is an IncFII(pHN7A8)/IncR-type multidrug-resistant plasmid with a length of 133,775 bp. It shares high homology with pKP1303-P2 and pKP1441-P2, showing over 99% coverage and no less than 99.79% identity.

The *NDM-1* gene is located on plasmid pKP1225-P3 (Fig. 4c), 102,184 bp in length. Analysis using Plasmid Finder did not identify any known replicon type for this plasmid. pKP1225-P3 is also highly conserved with pKP1303-P3 and pKP1441-P3, exhibiting 100% coverage and above 99.83% identity.

Further comparison with plasmid sequences in the NCBI database revealed that pKP1225-P2 is highly similar to 76 plasmids from *K. pneumoniae* (coverage ≥90%). Among these, it showed 100% query coverage and 99.98% identity with plasmid pXH1507-2 (GenBank: CP092795.1) from *K. pneumoniae* XH1507. In contrast, pKP1225-P3 displayed high homology only with plasmid pPL2335-*NDM* (GenBank: PQ279302.1) from *K. pneumoniae* PL2335 (100% coverage, 99.98% identity). Other *bla*_NDM-1_-carrying plasmids mostly showed less than 80% coverage and were derived from diverse bacterial species, including *Citrobacter, Enterobacter*, *Klebsiella*, and *Morganella*, among other gram-negative bacteria.

**Fig 5:**
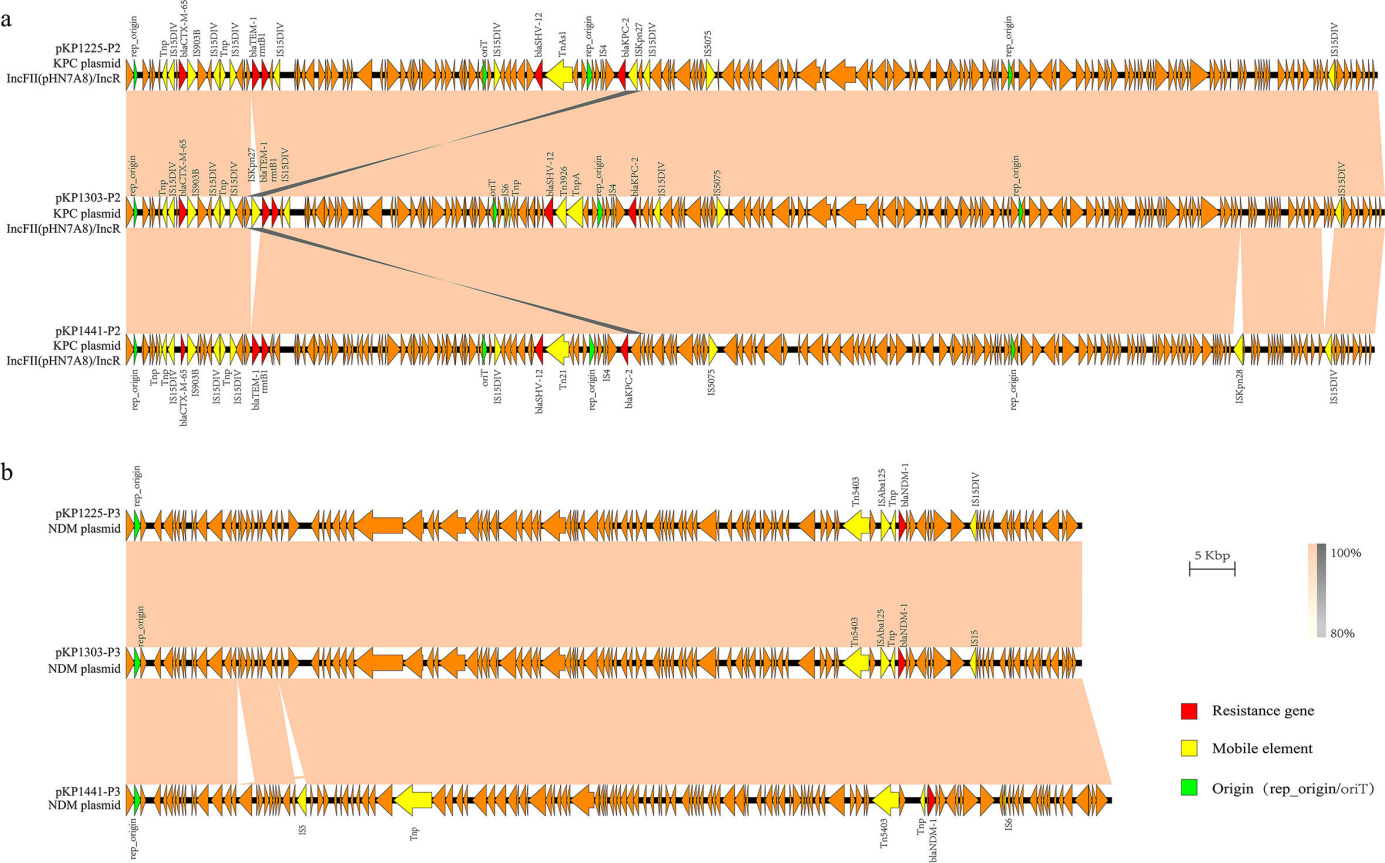
Co-linear alignment of plasmid genomes. (**a**) Co-linear genome alignment of the plasmid pKP1225-P2 with KP1303 and KP1441. (**b**) Co-linear genome alignment of the plasmid pKP1225-P3 with KP1303 and KP1441. Resistance genes are colored red, mobile elements are marked yellow, origins are labeled green, and homologous regions are shown in orange (standard sequences) or gray (inverted sequences) shading. Homology was defined as ≥80% identity. Alignments smaller than 1 kbp were removed.

Collinearity analysis of the plasmid genomes (Fig. 5) revealed that pKP1225-P2, pKP1303-P2, and pKP1441-P2 all carry identical replication origins and one transfer origin. The main structural difference in pKP1303-P2 involves the inversion and translocation of the insertion sequence ISKpn27 (belonging to the IS481 family). The core genetic context surrounding the *KPC-2* gene is structured as rep_origin–IS4–*bla*_KPC-2_–IS5075. In pKP1441-P3, the primary difference is the insertion of ISKpn26 (belonging to the IS5 family) within the region 18,255–19,235 bp.

Although the three strains clustered into different branches in the SNP-based phylogenetic tree, they are highly closely related phylogenetically and can be classified within the same clonal group. The fact that these strains carry identical or highly similar plasmids and share a common genomic background suggests that all 44 K2N1-CRKP isolates—including these three—may have originated from a single clonal transmission event.

**Fig 6:**
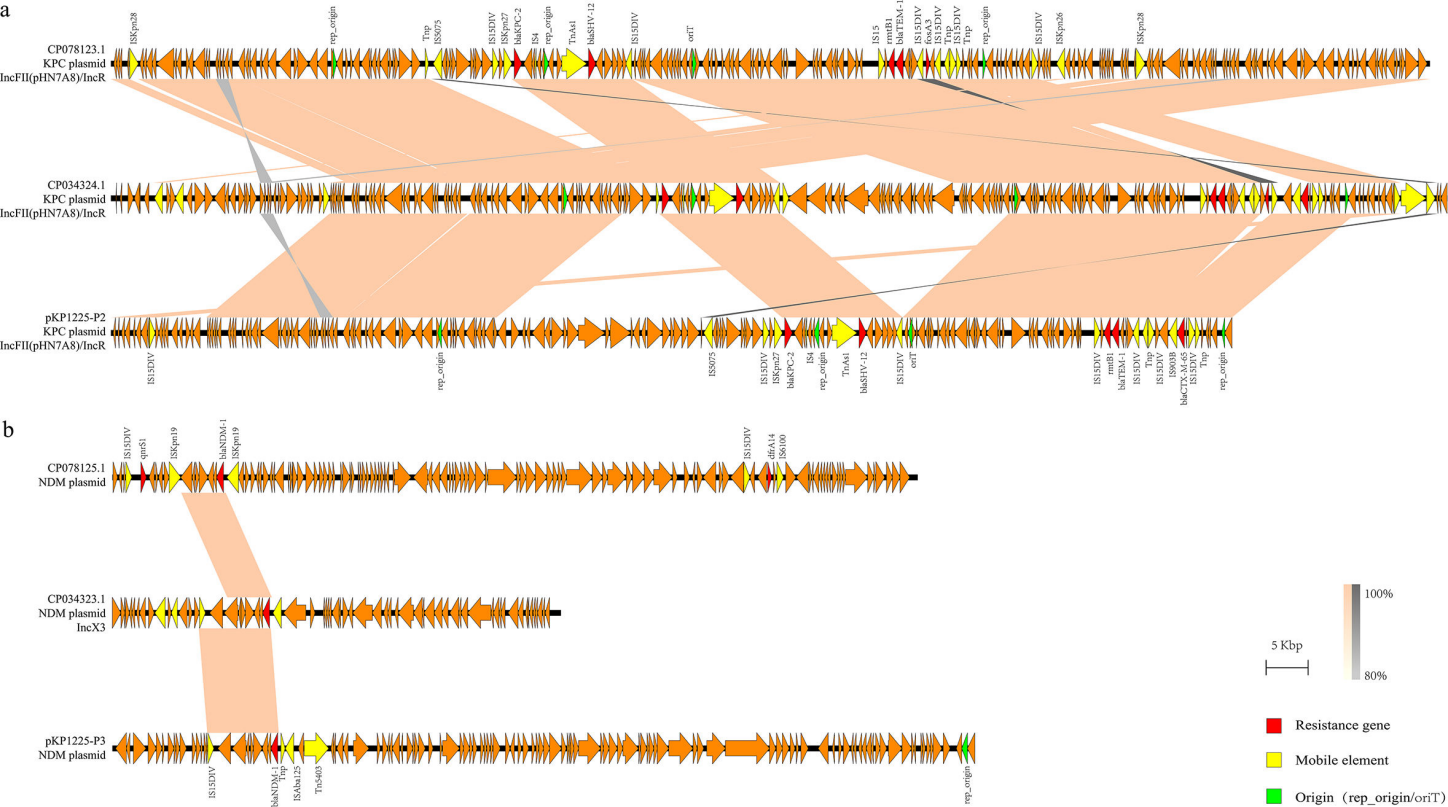
Co-linear alignment of plasmid genomes. (**a**) Co-linear genome alignment of the plasmid pKP1225-P2 with CP078123.1 and CP034324.1. (**b**) Co-linear genome alignment of the plasmid pKP1225-P3 with CP078125.1 and CP034323.1.

Further collinearity comparison between pKP1225-P2 and plasmids from two domestically reported strains (28, 29) (Fig. 6) showed coverages of 88% and 67% and identities of 99.92% and 99.98%, respectively, with the *bla*_KPC-2_-carrying plasmids (GenBank: CP078123.1/CP034324.1). For pKP1225-P3, comparisons with *bla*_NDM-1_-carrying plasmids (GenBank: CP078125.1/CP034323.1) yielded query coverages of 10% and 18% and identities of 100% and 99.88%, respectively. In the three isolates of this study, the *NDM-1* gene is consistently flanked by *ble*_MBL_ and a transposase gene, resembling the structures observed in plasmids GenBank CP078125.1 (*ble*_MBL_–*bla*_NDM-1_–ISKpn19) and CP026590.1.

### Assessment of bacterial fitness cost and plasmid stability

**Fig 7:**
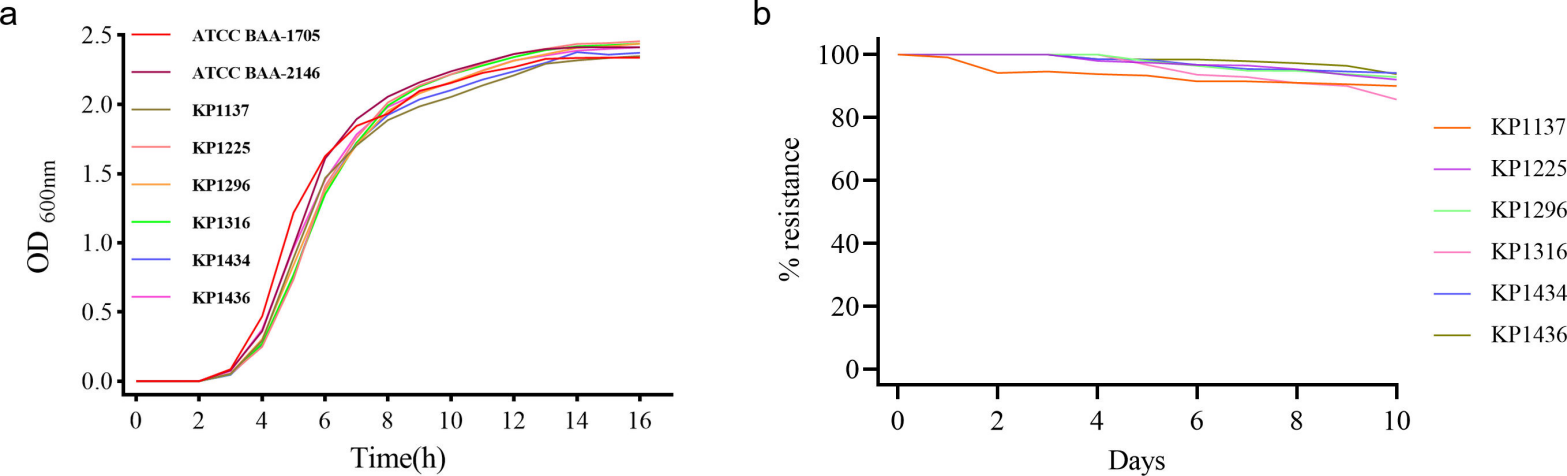
Growth curves and stability of KPC-2-NDM-1-CRKPs. (**a**) Growth curves of six KPC-2-NDM-1-CRKPs, quality control standard strain ATCC BAA-1705 (KPC-2), and ATCC BAA-2146 (NDM-1). (**b**) Stability of *bla*_KPC-2_ and *bla*_NDM-1_ plasmids along 10-day serial passage.

To evaluate whether CRKP strains co-harboring *bla*_KPC-2_ and *bla*_NDM-1_ incur a fitness cost due to the carriage of dual carbapenemase genes, we randomly selected six such strains (KP1137, KP1225, KP1296, KP1316, KP1434, and KP1436). We compared them with control strains: ATCC BAA-1705 (producing KPC-2) and ATCC BAA-2146 (producing NDM-1), using growth curve analysis (Fig. 7a). Interestingly, no significant difference in growth rate was observed between the six dual-positive strains and the single-carbapenemase-producing controls.

Notably, all 44 K2N1-CRKP strains isolated consecutively in 2021 exhibited a high degree of clonal relatedness, pointing to their substantial stability under actual clinical conditions. We conducted a 10-day serial passage experiment under antibiotic-free laboratory conditions to test this hypothesis and assess plasmid stability in the six selected strains (Fig. 7b). Results showed high plasmid retention rates in all strains: five isolates maintained retention above 90%, while KP1316 showed a retention rate of 85.7%. PCR confirmed these results.

Furthermore, we evaluated the antimicrobial resistance phenotypes of the passaged strains on day 1 and day 10 using meropenem and ceftazidime/avibactam disk diffusion tests (Table S1). All strains maintained stable resistance profiles, with no significant differences observed between the two time points, indicating that they can sustain their resistance without antibiotic pressure.

## DISCUSSION

Among the 44 patients infected with dual KPC-2-and NDM-1-producing CRKP included in this study, the vast majority (43/44, 97.73%) had undergone invasive procedures, including surgery, central venous catheterization, urinary tract catheterization, and endotracheal intubation. Among these, 70.45% (31/44) were complicated by severe ventilator-associated pneumonia (VAP). These findings suggest that invasive procedures significantly elevate the risk of infection with such resistant strains, consistent with previous reports on CRKP causing VAP (12).

Among the 26 fatal cases, 96.15% (25/26) had one or more underlying diseases, indicating that comorbidities may be an essential risk factor influencing patient outcomes. Such patients are generally immunocompromised, rendering them less capable of resisting bacterial infections. Moreover, K2N1-CRKP strains carrying a high load of virulence genes may possess enhanced invasiveness and transmission capacity, further aggravating the infection process. Therefore, greater clinical attention and more proactive multidisciplinary intervention strategies are warranted for patients with underlying diseases infected with K2N1-CRKP to improve their prognosis.

Furthermore, this study revealed apparent spatiotemporal clustering of these infections: 11 strains from the Neurological Intensive Care Unit (NICU) were detected within 2 months, 6 isolates from the Neurosurgery Department within 16 days, and another 4 strains from the Emergency Intensive Care Unit (EICU) within 1 month. These results suggest that K2N1-CRKP may have already undergone clonal spread within healthcare facilities, underscoring the urgent need for infection control departments to heighten vigilance and implement effective measures to prevent further dissemination.

All K2N1-CRKP isolates exhibited high-level resistance to penicillins, cephalosporins, and carbapenems and were generally resistant to ceftazidime/avibactam. Notably, strains KP1239 and KP1441 were resistant to tigecycline and polymyxin B, respectively, and the corresponding patients ultimately died. Although tigecycline and polymyxin B remain critical therapeutic options for infections caused by multidrug-resistant gram-negative bacteria, our results indicate that resistance to these agents has already emerged and may increase, accompanied by high mortality rates.

In terms of treatment, combination regimens such as aztreonam/avibactam or polymyxin B combined with meropenem, amikacin, and gentamicin may possess certain therapeutic potential against K2N1-CRKP infections (28, 29). We speculate that during the COVID-19 pandemic, the widespread use of antibiotics, co-infections with multiple microorganisms, and selective pressure from repeated bacterial colonization collectively facilitated the global dissemination of carbapenemase co-producing strains (30, 31). Furthermore, the coexistence of *bla*_KPC-2_ and *bla*_NDM-1_ may synergistically enhance the resistance level of bacterial strains to carbapenems and other antimicrobial agents (32).

In China, strains co-harboring *bla*_KPC-2_ and *bla*_NDM-1_ dominate among dual-carbapenemase-producing *K. pneumoniae*, with ST11 being the predominant ST, which is also the most prevalent ST among CRKP in China (3). Studies have indicated a distinct ST preference for *bla*_KPC-2_ and *bla*_NDM-1_ in CRKP: surveillance data from the carbapenem-resistant enterobacteriaceae (CRE) network show that 97.1% of ST11 and 74.2% of ST15 CRKP carry *bla*_KPC-2_, while *bla*_NDM-1_ is predominantly found in ST17 (91.4%) and ST895 (100%) (33). All 44 K2N1-CRKP isolates involved in this study were identified as ST11 by MLST, with capsular serotype KL64 and lipopolysaccharide antigen type O1/O2v1. PFGE revealed a homology of over 88% among the strains. These results consistently demonstrate that this group of strains belongs to an ST11-KL64 K2N1-CRKP clonal cluster that has propagated within the hospital environment, suggesting a likely common clonal origin.

Previous studies have shown that *bla*_KPC-2_ is typically located on IncFII-type plasmids, whereas *bla*_NDM-1_ is often found on other plasmid types (33). In this study, pKP1225-P2, pKP1303-P2, and pKP1441-P2 were all identified as IncFII(pHN7A8)/IncR-type multidrug-resistant plasmids, while pKP1225-P3, pKP1303-P3, and pKP1441-P3 could not be matched to any known replicon type via Plasmid Finder. Further comparisons revealed that plasmids similar to pKP1225-P3 carrying *bla*_NDM-1_ are present in gram-negative bacteria, such as *Citrobacter*, *Enterobacter*, *Klebsiella*, and *Morganella*. In contrast, plasmids highly similar to pKP1225-P2 were found only in *K. pneumoniae*. Based on these differences in host range, we hypothesize a potential evolutionary pathway: CRKP initially carrying only *bla*_KPC-2_ may have evolved into K2N1-CRKP by acquiring a class of highly transferable plasmids carrying *bla*_NDM-1_. Existing reports support this hypothesis: conjugation experiments have indicated that *bla*_NDM-1_-carrying plasmids generally exhibit higher conjugation transfer efficiency than those carrying *bla*_KPC-2_. Phylogenetic analyses also show that K2N1-CRKP strains are more closely related to *K. pneumoniae* carrying *bla*_KPC-2_ plasmids than strains harboring *bla*_NDM-1_ plasmids (33). Additionally, some studies have reported the emergence of IncFII/IncR fusion plasmids co-harboring *bla*_KPC-2_ and *bla*_NDM-1_ in clinical hypervirulent ST11-KL64 CRKP isolates (34). These findings suggest that *bla*_NDM-1_-carrying plasmids demonstrate high transferability within bacterial species and possess broad potential for interspecies dissemination. More notably, *bla*_KPC-2_ and *bla*_NDM-1_ coexisting on a single plasmid have already emerged, further exacerbating the risk of coordinated resistance gene transmission.

Existing studies indicate that interactions between different plasmids within bacterial cells can enhance strain fitness, replication efficiency, and potential for horizontal gene transfer (35, 36). Among these, IS26-mediated plasmid fusion events play a key role in the dissemination of resistance in *K. pneumoniae*, significantly increasing the risk of evolving multidrug resistance and helping to expand the host range of plasmids (37, 38). In the plasmid pKP1441-P3 identified in this study, we also detected an IS, ISKpn26, belonging to the IS5 family. This structural feature highlights the need for increased vigilance regarding the potential risks of disseminating fusion plasmids involving genomic recombination and conjugative transfer.

WGS revealed that pKP1225-P1 carries multiple virulence genes, including *rmpA*, *rmpA2*, *rmpC*, *iucABCD*, *iutA*, *iroN*, and *peg-344*. Current studies suggest that specific genes located on virulence plasmids—such as *rmpA*, *rmpA2*, *iucA*, *iroB*, and *peg-344*—are considered the most accurate molecular markers for defining hvKP (39). Based on this criterion, 35 isolates co-harboring *iucA* and *rmpA/rmpA2* were identified in this study as CR-hvKP. Among these, *rmpA* and *rmpA2* enhance mucoviscosity and pathogenicity and are closely associated with hypercapsule production, a well-established key virulence factor in hvKP (40, 41). Additionally, aerobactin, encoded by the *iucABCD* gene cluster with its receptor expressed by *iutA*, is regarded as a crucial siderophore system in hvKP. It is described in over 90% of hvKP strains but only about 6% of cKP (41). Studies have shown that pLVPK-like virulence plasmids can transfer from hvKP to CRKP and even to *Escherichia coli* strains with the assistance of self-transmissible IncF plasmids and inappropriate antibiotic use (42). During this process, some virulence genes may be lost (42). Notably, none of the strains in this study expressed aerobactin, suggesting that these CR-hvKP isolates may have acquired partial virulence plasmids and genes from hvKP on a CRKP genetic background. Furthermore, all strains carried 11 virulence genes related to the T6SS, indicating potential enhanced biofilm formation, elevated resistance levels, and increased virulence (43).

*G. mellonella* larvae are an important *in vivo* model for studying *K. pneumoniae* infection processes, host antioxidant responses, and the extent of cellular damage. This model is also suitable for AST and investigating host interactions with resistant *K. pneumoniae* (44). In this study, we evaluated the virulence of three isolates using the *in vivo G. mellonella* infection model and *in vitro* serum killing assays. The results showed that KP1225 was resistant to healthy human serum (Grade 6) and caused a significant reduction in the survival of infected larvae. Interestingly, KP1303 exhibited discordance between its *in vivo* and *in vitro* virulence phenotypes—a phenomenon that is not unique and has been reported in the literature (12, 45). The underlying mechanisms for this discrepancy remain unclear. Moreover, the significant heterogeneity in virulence observed among the three representative isolates, spanning high, intermediate, and low pathogenicity in both serum killing assays and *G. mellonella* models, highlights that hypervirulence is not a uniform trait within the ST11-KL64 clone. Our integrated genomic and phenotypic analysis of these carefully selected strains confirms that even closely related isolates can exhibit divergent pathogenic potential, a finding with crucial implications for patient risk stratification.

Throughout the 10-day serial passage, the carbapenemase genes *KPC-2* and *NDM-1* remained stable in all six K2N1-CRKP strains. This result is consistent with those of previous studies (33, 37), further underscoring the importance of effective control measures to limit the spread of such strains. Compared to CRKP, producing only a single carbapenemase, these six dual-producing strains showed no significant difference in growth rate, indicating that they can stably maintain both *bla*_KPC-2_ and *bla*_NDM-1_ long-term without a substantial fitness cost. This finding heightens concern regarding K2N1-CRKP, as they are challenging to treat clinically and exhibit stable growth and persistent carriage of resistance genes, making the loss of these genes highly unlikely.

In this study, 44 K2N1-CRKP strains were detected within a single year. These isolates demonstrated high homology, and most exhibited both high virulence and phenotypic stability, providing further insights into the clinical characteristics, resistance mechanisms, virulence profiles, transmission potential, and evolutionary trends of ST11-KL64 K2N1-CR-hvKP. Notably, at least 35 ST11-KL64-K2N1-CR-hvKP strains carried additional virulence and resistance genes, indicating a concerning trend of convergent evolution between carbapenem resistance and hypervirulence within already widespread *K. pneumoniae* lineages.

Comprehensive prevention and control strategies are urgently needed to address the challenges posed by the spread of K2N1-CR-hvKP. First, clinically effective and urgent prevention, surveillance, diagnostic, and treatment systems should be established, particularly for patients with underlying diseases, to curb the transmission of such clones. Second, given the high transferability of *bla*_NDM-1_-carrying plasmids, it is recommended that all CRKP-infected patients treated with ceftazidime/avibactam or carbapenems undergo carbapenemase genotyping, especially in cases of mixed bacterial co-infections. Finally, developing novel antibacterial agents is necessary to expand effective clinical treatment options as resistance patterns evolve.

Although this study successfully identified a collection of 44 ST11-KL64-K2N1-CRKP strains—at least 35 of which can be defined as CR-hvKP—several limitations should be noted. First, the retrospective study design limited our ability to obtain more detailed clinical information or retrospectively collect environmental samples, thereby hindering precise tracing of the clone’s transmission routes. Additionally, since virulence assays and third-generation sequencing were performed on only three isolates, the results may not fully represent the genetic and phenotypic diversity of all 44 strains.

### Conclusion

The findings of this study demonstrate that ST11-KL64-K2N1-CR-hvKP strains in Chinese hospital settings can co-harbor *bla*_KPC-2_ and *bla*_NDM-1_ without significant fitness costs and are capable of stable clonal dissemination. The convergence of hypervirulence with carbapenem resistance and even with resistance to polymyxin B and tigecycline poses a serious challenge to clinical infection management. Therefore, this study underscores the urgency and importance of sustained surveillance and effective infection control strategies against the spread and potential clonal dissemination of K2N1-CRKP, particularly of K2N1-CR-hvKP strains.

## Data Availability

The data that support the findings of this study can be found in the NCBI Genbank repositories under the Bioproject PRJNA1292292, PRJNA1292749, and PRJNA1292752.

## SUPPLEMENTAL MATERIAL

Figure S1 spectrum.02911-25-s0001.pdf Gantt chart bacterial isolates by ward and time.

Table S1 spectrum.02911-25-s0002.pdf The results of MEM and CZA sensitivity of six strains by the KB method.

ASM does not own the copyrights to Supplemental Material that may be linked to, or accessed through, an article. The authors have granted ASM a non-exclusive, world-wide license to publish the Supplemental Material files. Please contact the corresponding author directly for reuse.
