# Supplementary material for: Clinical characteristics and molecular evolution of ST11-KL64 carbapenem-resistant hypervirulent Klebsiella pneumoniae co-producing KPC-2 and NDM-1 from China
Source: Microbiol Spectr. 2026 Feb 9;14(3):e02911-25. doi: 10.1128/spectrum.02911-25 (PMC12955449; doi:10.1128/spectrum.02911-25)
Supplement: Figure S1 — Gantt chart bacterial isolates by ward and time. [file spectrum.02911-25-s0001.pdf]

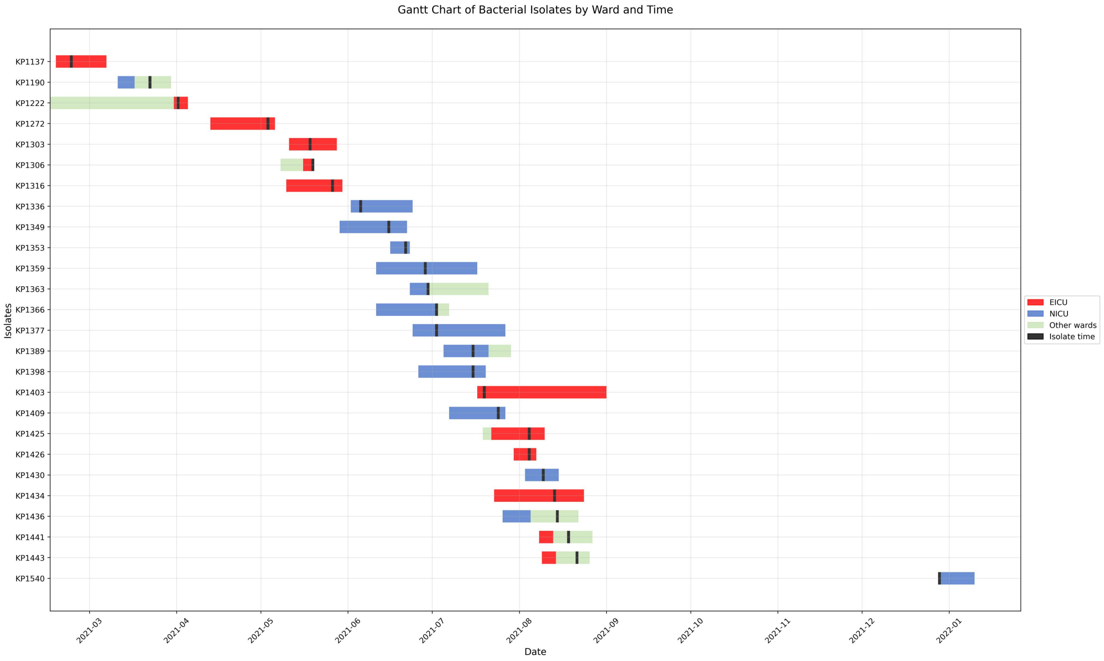

Figure S1. Gantt chart bacterial isolates by ward and time.

EICU cases are colored red, NICU cases are marked blue, the other ward cases are labeled cyan, and the strain isolation times are shown in black.
