# Supplementary material for: Clinical characteristics and molecular evolution of ST11-KL64 carbapenem-resistant hypervirulent Klebsiella pneumoniae co-producing KPC-2 and NDM-1 from China
Source: Microbiol Spectr. 2026 Feb 9;14(3):e02911-25. doi: 10.1128/spectrum.02911-25 (PMC12955449; doi:10.1128/spectrum.02911-25)
Supplement: Table S1 — The results of MEM and CZA sensitivity of six strains by the KB method. [file spectrum.02911-25-s0002.pdf]

Table S1. The results of MEM and CZA sensitivity of six strains by KB method

	MEM(mm)		CZA(mm)	
	d1	d10	d1	d10
KP1137	6	6	15	15
KP1225	6	6	14	14
KP1296	6	6	15	15
KP1316	6	6	15	15
KP1434	6	6	15	15
KP1436	6	6	14	14

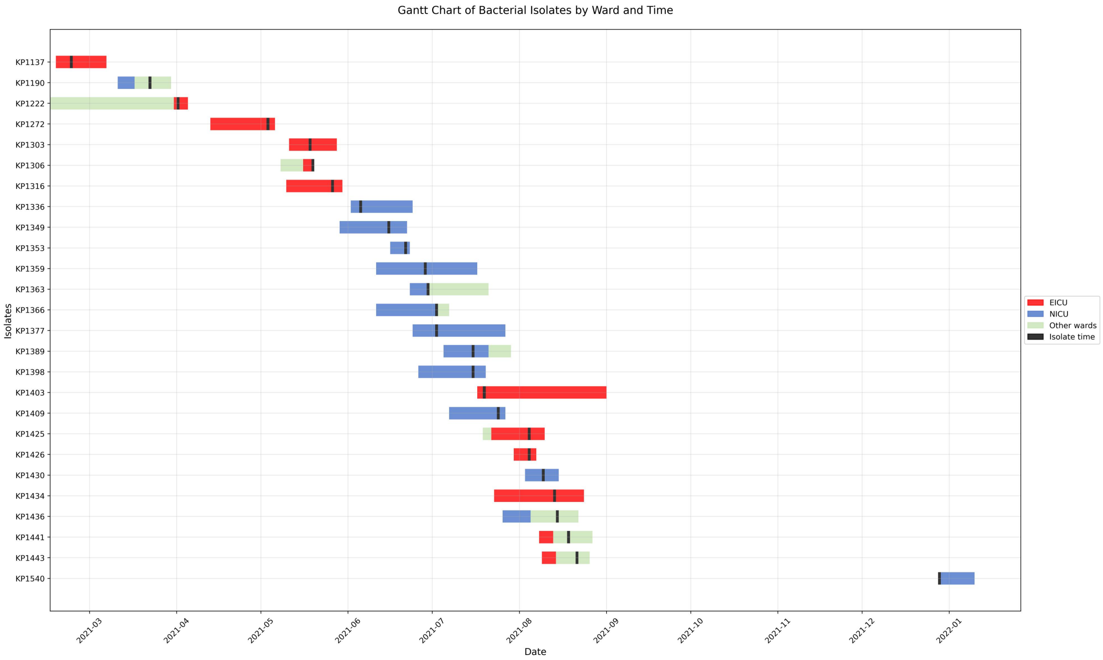

Figure S1. Gantt chart bacterial isolates by ward and time.

EICU cases are colored red, NICU cases are marked blue, the other ward cases are labeled cyan, and the strain isolation times are shown in black.
